# Occurrence of septuple and elevated *Pfdhfr*-*Pfdhps* quintuple mutations in a general population threatens the use of sulfadoxine-pyrimethamine for malaria prevention during pregnancy in eastern-coast of Tanzania

**DOI:** 10.1186/s12879-020-05253-7

**Published:** 2020-07-22

**Authors:** George M. Bwire, Wigilya P. Mikomangwa, Manase Kilonzi

**Affiliations:** 1 Department of Pharmaceutical Microbiology, Muhimbili University of Health and Allied Sciences, P.O. Box 65013, Dar es Salaam, Tanzania; 2 Department of Clinical Pharmacy and Pharmacology, Muhimbili University of Health and Allied Sciences, P.O. Box 65013, Dar es Salaam, Tanzania

**Keywords:** *Pfdhfr*, *Pfdhps*, Sulfadoxine-pyrimethamine, Septuple, Quintuple, Tanzania

## Abstract

**Background:**

*Plasmodium falciparum* dihydrofolate reductase (*Pfdhfr*) and dihydropteroate synthetase (*Pfdhps*) mutations compromise the effectiveness of sulfadoxine-pyrimethamine (SP) for treatment of uncomplicated malaria, and are likely to impair the efficiency of intermittent preventive treatment during pregnancy (IPTp). This study was conducted to determine the level of *Pfdhfr-Pfdhps* mutations, a decade since SP was limited for IPTp use in pregnant women in Tanzania.

**Methods:**

*P. falciparum* genomic DNA was extracted from dried blood spots prepared from a finger prick. Extracted DNA were sequenced using a single MiSeq lane by combining all PCR products. Genotyping of *Pfdhfr* and *Pfdhps* mutations were done using bcftools whereas custom scripts were used to filter and translate genotypes into SP resistance haplotypes.

**Results:**

The *Pfdhfr* was analyzed from 445 samples, the wild type (WT) *Pfdhfr* haplotype NCSI was detected in 6 (1.3%) samples. Triple *Pfdhfr***IRN**I (mutations are bolded and underlined) haplotype was dominant, contributing to 84% (number [n] = 374) of haplotypes while 446 samples were studied for *Pfdhps*, WT for *Pfdhps* (SAKAA) was found in 6.7% (*n* = 30) in samples. Double *Pfdhps* haplotype (S**GE**AA) accounted for 83% of all mutations at *Pfdhps* gene. Of 447 *Pfdhfr-Pfdhps* combined genotypes, only 0.9% (*n* = 4) samples contained WT gene (SAKAA-NCSI). Quintuple (five) mutations, S**GE**AA-**IRN**I accounted for 71.4% (*n* = 319) whereas 0.2% (*n* = 1) had septuple (seven) mutations (**AG**K**GS**-**IRN**I). The overall prevalence of *Pfdhfr* K540E was 90.4% (*n* = 396) while *Pfdhps* A581G was 1.1% (*n* = 5).

**Conclusions:**

This study found high prevalence of *Pfdhfr*–*Pfdhps* quintuple and presence of septuple mutations. Mutations at *Pfdhfr* K540E and *Pfdhps* A581G, major predictors for IPTp-SP failure were within the recommended WHO range. Abandonment of IPTp-SP is recommended in settings where the *Pfdhfr* K540E prevalence is > 95% and *Pfdhps* A581G is > 10% as SP is likely to be not effective. Nonetheless, saturation in *Pfdhfr* and *Pfdhps* haplotypes is alarming, a search for alternative antimalarial drug for IPTp in the study area is recommended.

## Background

Antimalarial drugs use and vector control are the commendable tools for global malaria prevention and control respectively [1]. However, resistance of malaria parasites to antimalarial drugs and resistance of Anopheles mosquitoes to insecticides impair the progressive fight against malaria [2]. Resistance of *Plasmodium falciparum* to previous generations of medicines, i.e., chloroquine (CQ) [3] and sulfadoxine-pyrimethamine (SP) [4] influenced the replacement of CQ with SP [5], then artemisinin-based combination therapy (ACT) as the first-line treatment for uncomplicated malaria in Tanzania [6].

The changes in malaria treatment policy were supported by evidence from molecular epidemiological resistance surveillance against SP [7], mainly *P. falciparum* dihydrofolate reductase (*Pfdhfr*) and dihydropteroate synthetase (*Pfdhps*) enzymes mutations [8, 9]. Mutations in the *Pfdhps* gene at codons serine 436 to alanine (S436A), alanine 437 to glycine (A437G), lysine 540 glutamic acid (K540E), alanine 581 to glycine (A581G) and alanine 613 to serine (A613S) predicts sulfadoxine resistance whereby mutations in the *Pfdhfr* gene at codons cysteine 50 to arginine (C50R), asparagine 51 to isoleucine (N51I), cysteine 59 to arginine (C59R), Serine 108 to asparagine/threonine (S108 N/T), and isoleucine 164 to leucine (I164L) are associated with pyrimethamine resistance [10].

Mutations in both the *Pfdhfr/Pfdhps* genes which greatly influence SP clinical treatment failures [11] resulted SP being unfit for first -line treatment of uncomplicated malaria in Tanzania [12]. However, in 2006 the drug was limited for intermittent preventive treatment during pregnancy (IPTp-SP), in areas with moderate to high malaria transmission [13]. Pregnant women regardless of the presence or absence of malaria should administer at least three curative doses of SP with an interval of at least 1 month between the two doses, starting in the second trimester of pregnancy [14], for preventing pregnancy associated malaria and improve pregnancy outcomes [13].

The elevated *Pfdhfr/Pfdhps* protein mutations and saturation of genetic haplotypes such as quintuple (CIRNI-SGEAA) and sextuple (CIRNI-SGEGA) reported in Tanzania [10] pose risks against the effectiveness of SP in preventing placental malaria during pregnancy. In this regard, World Health Organization (WHO) recommends abandonment of IPTp-SP in settings where the *Pfdhfr* K540E prevalence is > 95% and *Pfdhps* A581G is > 10% because SP is more likely to be less effective [15].

For the purpose of guiding IPTp-SP policy and inform on the current SP resistance status, routinely surveillance of SP effectiveness using molecular markers should be implemented as recommended by WHO [15]. Therefore, a study was conducted in a general population to determine the status of mutations associated with SP resistance, more than one decade since SP was limited for IPTp use in pregnant women in Tanzania.

## Methods

### Study design

A hospital based surveillance of molecular markers for SP resistance was conducted for 4 months from April, 2019 at Kibiti Health Center (KHC) found in Eastern part of Tanzania. The study was conducted to determine the current antimalarial status of SP, more than a decade since SP were limited for IPTp for pregnant women in Tanzania. Tanzania is found in sub-Saharan region where malaria is endemic, adopted the use of SP for malaria prevention in pregnancy women for more than one decade [12].

### Study area

KHC is found in Kibiti District, Pwani region. Pwani region is one of the five regions found along the coastal of Indian Ocean. Pwani region is bordered to north by Tanga region, to the east by Dar es Salaam region and the Indian Ocean, to the south by the Lindi region, and to the west by the Morogoro region. The coastal region experiences malaria transmission throughout the year with regional prevalence ranging between 3.1 and 14.8% [16].

### Study population

Patients attending clinic at KHC who presented with malaria infection related symptoms qualified for inclusion in the study. The suggestive symptoms example fever, general body weakness and headache were confirmed by the attending clinician [17]. Rapid malaria testing was performed using CareStart™ malaria HRP2/pLDH (Pf/pan) Combo test (Access Bio, Ethiopia). Samples tested positive by rapid tests qualified for blood smear (BS) microscopy for confirmation. A total of 489 positive BS microscopy qualified for further dried blood spot preparation.

### DBS preparation and DNA extraction

Dried Blood Spots (DBS) were prepared as per MalariaGEN SpotMalaria, DBS collection protocol [18]. Briefly, a well sterilized middle patient’s finger was pricked, and 4 blood spots from each patient were dropped on a separate circle of the filter paper; two on each paper card. The spotted blood were dried and put in the desiccant sachet for storage. DNA from the DBS was extracted following QIAamp DNA Investigator Kit for isolation of total DNA from filter papers (Qiagen, Valencia, CA, USA) and as described by Oyola et al.,2016 [19].

### Genotyping of *Pfdhfr* and *Pfdhps* mutations

Molecular genotyping of *Pfdhfr* and *Pfdhps* gene mutations associated with SP-resistance was performed by Wellcome Sanger Institute, UK as previously described but customized for *Pfdhfr* and *Pfdhps* mutations [20].

To summarize, targeted genotypes were spotted and multiplex PCR primers were designed using mPrimer software [21] and were run according to Wellcome Sanger Institute protocol (Goncalves, manuscript in preparation; https://www.malariagen.net/projects/spotmalaria). Primers were made to amplify products between 190 and 250 bp and combined into 3 pools. A multi steps protocol was used to first amplify the target regions of the *P. falciparum* genome, then PCR to combine sequencing and multiplexing adapters. About 384 samples were sequenced in one MiSeq lane by combining all PCR products. Samples were de-plexed using the multiplexing adapters and individual CRAM files were aligned to a modified amplicon reference genome for further analysis.

### Statistical analysis

Statistical Package for Social Sciences version 25 (SPSS Software, Chicago Inc., USA) was used to import laboratory data from the Microsoft Excel sheet (Redmond, WA) for data analysis and interpretation. Genotyping was done using bcftools and custom scripts to filter and translate genotypes into *Pfdhfr* and *Pfdhps* resistance haplotypes. Haplotypes were expressed as frequencies and percentages.

## Results

### Haplotypes for *Pfdhfr* and *Pfdhps*

The *Pfdhfr* was analyzed from 445 samples, the wild type (WT) *Pfdhfr* haplotype NCSI was detected in only 1.3% (number [n] = 6) samples. Triple *Pfdhfr***IRN**I haplotype was dominant, contributing to 84% (*n* = 374) of haplotypes. The total of 446 samples were studied for *Pfdhps*. The WT for *Pfdhps* was found in 6.7% (*n* = 30) of all samples detected. The most common mutation was the change of amino acid alanine to glycine (A437G) and lysine to glutamic acid (K540E). Double *Pfdhps* haplotype (S**GE**AA) accounted for 83% of mutations of the *Pfdhps* gene (Table 1). The overall prevalence of K540E was 90.4% (*n* = 396) while A581G was 1.1% (*n* = 5).

**Table 1:** *Pfdhfr* and *Pfdhps* haplotypes

Gene	Haplotype	Frequency (n)	Percentage (%)
***Pfdhfr*(*n*** **= 445)**	NCSI	6	1.3
	I***	1	0.2
	**I**C**N**I	30	6.7
	**I**NI*	5	1.1
	IR**	5	1.1
	**IRN**I	374	84.0
	NI**	1	0.2
	NR**	1	0.2
	N**RN**I	22	4.9
***Pfdhps*(*n*** **= 446)**	SAKAA	30	6.7
	**A**AAA*	1	0.2
	**A**AKAA	6	1.3
	**AGE**AA	6	1.3
	**AG**K**GS**	1	0.2
	**FG**KAA	1	0.2
	**GE**AA*	2	0.4
	**H**AKAA	2	0.4
	SAAA*	1	0.2
	SA**E**AA	3	0.7
	S**E**AA*	3	0.7
	SG***	2	0.4
	S**G**AA*	5	1.1
	SGE**	6	1.3
	S**GE**A*	1	0.2
	S**GE**AA	370	83.0
	S**GEG**A	4	0.9
	S**GK**AA	2	0.4

*Indicates missing genotype(s) during sequencing. Each letter represents an amino acid, *A* alanine, *C* cysteine, *E* Glutamic acid, *F* Phenylalanine, *G* Glycine, *H* Histidine, *I* Isoleucine, *K* Lysine, *N* Asparagine, *R* arginine, *S* Serine. Bold underline represent amino acid changes. SAKAA and NCSI represent the wildtype (WT) amino acids for *Pfdhps* and *Pfdhfr*, respectively

### Combined *Pfdhfr* and *Pfdhps* haplotypes

Overall 447 (91.4%) genotypes were detected from 489 sequenced samples. The concomitant mutations (*Pfdhfr*-*Pfdhps*) were present in 99.1% (*n* = 443) of samples. Of 447 genotypes, only 4 samples (0.9%) were WT (SAKAA-NCSI). Concomitant mutations with quintuple haplotype (S**GE**AA-**IRN**I) dominated by 71.4% whereby sextuple haplotype (**AG**K**GS**-**IRN**I) was detected in one sample (0.2%) (Table 2).

**Table 2:** Combined *Pfdhfr-Pfdhps* haplotypes

No. of ***Pfdhps- Pfdhfr*** mutations (haplotype where applicable)	Frequency (n)	Percentage (%)
Wild type (SAKAA-NCSI)	4	0.9
Single (1)^a^	0	0
Double (2)^a^	11	2.5
Triple (3)^a^	30	6.7
Quadruple (4)^a^	73	16.3
Quintuple (5) (S**GE**AA-**IRN**I)	319	71.4
Sextuple (6)^a^	9	2.0
Septuple (7) (**AG**K**GS**-**IRN**I)	1	0.2
Total	447	100

^a^The haplotype(s) type were not indicated if the mutation category had multiple haplotypes

## Discussion

This study was conducted to determine the current SP resistance status, more than a decade since SP was limited for IPTp use in pregnant women in Tanzania.

The study found high level (99.1%) concomitant mutations (*Pfdhfr*-*Pfdhps*) where quintuple mutation (S**GE**AA-**IRN**I) dominated by 71.4%. Additionally, one sample detected septuple mutation (**AG**K**GS**-**IRN**I) and only 0.9% contained the WT (SAKAA-NCSI) protein. These results were close to the findings from the previous study which reported high-level of *P. falciparum* SP resistance with concomitant occurrence of septuple haplotype [10]. There was a high number of quintuple (S**GE**AA-**IRN**I) and few sextuple mutations. These haplotypes have been highly associated with sub-optimal IPTp-SP effectiveness in study conducted between 2008 and 2010 in Korogwe district, Tanzania [22]. Moreover, ﻿ high prevalence of the *Pfdhfr/Pfdhps* sextuple haplotype was associated with reduced birth- weight [23–25].

In this study, triple *Pfdhfr***IRN**I haplotype mutation was dominant, contributing to 84% of all *Pfdhfr* haplotypes whereas double *Pfdhps* haplotype (S**GE**AA) accounted for 83% of mutations of the *Pfdhps* haplotypes. Compared to study by Baraka et al.,2015 [10], *Pfdhfr***IRN**I haplotype were predominant in all sites with significantly higher frequencies at Muheza (93.3%) compared to Muleba (75.0%) and Nachingwea districts (70.6%). In this regard, there was 13.4% increase in *Pfdhfr***IRN**I haplotype prevalence when Nanchingwea (70.6%) and Kibiti district (84%) both found along the coastal region were compared. Additionally, the previous study found that double *Pfdhps* haplotype S**GE**AA was significantly high at Muheza (27.2%) and Muleba (20.8%) while none (0%) was detected at Nachingwea while the current study at Kibiti district detected 83% *Pfdhps* S**GE**AA mutations. Meaning that, from 2015 to 2019 there is an increase of 0 to 83% *Pfdhps* S**GE**AA mutation in the coastal region, respectively.

WHO malaria report of 2019 has clearly mentioned pregnant women as the most affected group and use of effective IPTp should be highly implemented to combat risks associated with pregnancy malaria [26]. On top of that, a recent study by Mikomangwa et al., [27] concluded that pregnant woman who was malaria positive had 11 times more risk of giving birth to low birth weight (LBW) child when compared to those who tested malaria negative.

Nevertheless, *Pfdhfr* K540E and *Pfdhps* A581G, major predictors for IPTp-SP failure, had the prevalence of 90.4% for *Pfdhfr* K540E and 1.1% for *Pfdhps* A581G. This guarantee the continued use of IPTp-SP in the study area. WHO recommends stoppage of SP for IPTp if *Pfdhfr* K540E prevalence > 95% and *Pfdhps* A581G > 10% [15]. However, rapid increase and saturation in *Pfdhfr* and *Pfdhps* mutations with spread of sextuples and septuple is of great concern. The effort to search for alternative drug (s) for IPTp in the study areas should be prioritized. The current study was conducted in the general population, hence limited data on clinical, birth outcomes and malaria infection status in pregnant women in relation to SP resistance genotypes.

## Conclusions

This study found high prevalence of *Pfdhfr*–*Pfdhps* quintuple mutations. Mutations at *Pfdhfr* K540E and *Pfdhps* A581G, major predictors for IPTp-SP failure were within the recommended WHO range. Abandonment of IPTp-SP is recommended in areas where the *Pfdhfr* K540E prevalence is > 95% and *Pfdhps* A581G is > 10% because SP is likely to be not effective. Saturation in *Pfdhfr* and *Pfdhps* haplotypes is alarming, a search for alternative antimalarial drug for IPTp, the study area in particular is recommended.

## Data Availability

The datasets generated and/or analysed during the current study are available from the corresponding author on reasonable request.

## Abbreviations

SP: Sulfadoxine-pyrimethamine
WT: Wild type
IPTp: Intermittent preventive treatment during pregnancy
*Pfdhfr*: *Plasmodium falciparum* dihydrofolate reductase
*Pfdhps*: *Plasmodium falciparum* dihydropteroate synthetase
WHO: World Health Organization
